# Distinction of Chaos from Randomness Is Not Possible from the Degree Distribution of the Visibility and Phase Space Reconstruction Graphs

**DOI:** 10.3390/e26040341

**Published:** 2024-04-17

**Authors:** Alexandros K. Angelidis, Konstantinos Goulas, Charalampos Bratsas, Georgios C. Makris, Michael P. Hanias, Stavros G. Stavrinides, Ioannis E. Antoniou

**Affiliations:** 1 Department of Mathematics, Aristotle University of Thessaloniki, 54124 Thessaloniki, Greece; kgoulasz@math.auth.gr (K.G.); geormakr@csd.auth.gr (G.C.M.); iantonio@math.auth.gr (I.E.A.); 2 Department of Information and Electronic Engineering, International Hellenic University, 57400 Thessaloniki, Greece; cbratsas@iee.ihu.gr; 3 Department of Physics, International Hellenic University, 65404 Kavala, Greece; mhanias@physics.ihu.gr (M.P.H.); s.stavrinides@ihu.edu.gr (S.G.S.)

**Keywords:** chaos, randomness, time series, network theory, visibility graph, phase space reconstruction network

## Abstract

We investigate whether it is possible to distinguish chaotic time series from random time series using network theory. In this perspective, we selected four methods to generate graphs from time series: the natural, the horizontal, the limited penetrable horizontal visibility graph, and the phase space reconstruction method. These methods claim that the distinction of chaos from randomness is possible by studying the degree distribution of the generated graphs. We evaluated these methods by computing the results for chaotic time series from the 2D Torus Automorphisms, the chaotic Lorenz system, and a random sequence derived from the normal distribution. Although the results confirm previous studies, we found that the distinction of chaos from randomness is not generally possible in the context of the above methodologies.

## 1. Introduction

Chaos was discovered by Poincaré by the end of the 19th century as non-analyticity and dynamical instability in the three-body problem, while the computational limitations arising from chaos were discovered by Lorenz [1] and were described as the “Butterfly Effect”. Chaos has been found in most mathematical models involving applications, like physics, engineering, biology, economics, medicine, sociology, geology, and astronomy. The main feature of chaotic systems, that of the sensitive dependance on initial conditions, means that very small variations in initial conditions can lead to very large, dramatic, and effectively unpredictable variations of the evolving trajectories. As a result, chaotic systems cannot be predicted and controlled systematically, although the trajectories are mathematically unique. In other words, the deterministic trajectories are not effectively determinable. This behavior is the essence of deterministic chaos, or simply chaos [1,2,3,4,5]. Chaos in time series refers to the presence of unpredictable, seemingly random behavior in data. This can occur when the underlying system generating the time series is highly sensitive to initial conditions, leading to complex behavior that is difficult to model or predict.

Chaos is statistically indistinguishable from randomness. This fact has been confirmed mathematically in terms of positive entropy production [6,7], as well as by difficulties in applying statistical methods [8,9]. Among others, chaos is the mathematical mechanism for random number generation [10,11].

The possibility to distinguish chaos from randomness should be based on algebraic and/or topological arguments as statistical analysis fails on this task [6,7,8,9]. A way that has been proposed to achieve this distinction is to transform a time series into a complex network and investigate whether this distinction can be achieved using network theory. In recent years, network-based approaches have emerged as powerful tools to unravel the underlying structure and dynamics of complex systems [12,13,14,15]. The goal of this work is to evaluate and compare the visibility and phase space methods for distinguishing chaos from randomness.

Visibility methods construct a visibility graph from a time series considering each data point as a node in a network. Two nodes are connected by an edge if they obey a certain visibility criterion, which is defined according to the chosen method. In this way, one constructs the visibility network associated with the topology of the time series, and this allows the system analysis from a network science viewpoint. A handful of approaches applying the property of visibility on the points of a time series have been proposed [14]. The main two methods are the Natural Visibility Graph (NVG) and the Horizontal Visibility Graph (HVG) [16,17]. Both are aiming to extract the dynamical properties of the corresponding systems by correlating the points of the studied time series. The structure and the dynamics of the time series are claimed to be preserved in the graph topology. Moreover, it has been claimed that the discrimination between deterministic chaos and stochasticity for the studied system can be achieved through the calculation of the degree distribution of the emerging network, by studying the time series of a state variable of the system [16,17].

Phase space reconstruction involves converting a time series into a graph by reconstructing the phase space of the system from its time series data. The resulting phase space is a higher dimensional space that encompasses all the potential states of the system, where each point signifies a unique state. The system’s dynamics can be portrayed as a trajectory within this phase space. Phase space reconstruction entails the creation of a graph representation of phase space, in which the nodes symbolize the states of the system and the edges depict the transitions between these states. This facilitates the analysis of chaotic system behavior over time and the identification of patterns and trends within the data. This way allows to calculate typical metrics like correlation dimension and Kolmogorov entropy. On the other hand, according to Provenzale et al. [18], distinguishing between low dimensional chaos and any correlated noise should not be based solely on correlation dimension estimates within the phase space, since there have been reported cases of other types of stochastic processes mimicking the properties of low dimensional chaos, even in the case of infinite data sets [19]. Considering this limitation, other approaches have been proposed like the nonlinear analysis of the first differences or their equivalent, i.e., the numerical approximation of the derivative of the studied time series. In this case coincidence in the calculation of the correlation dimension between the initial time series and its first differences, provide a trustworthy conclusion of the deterministic or stochastic nature of the studied time series [20]. Finally, it should be mentioned that the deterministic or stochastic nature of a system is decided by combining various metrics, according to different methodologies [21]. In an attempt to exploit the emergence of properties of deterministic chaotic systems, the Phase Space Network has been proposed. According to this method, each point of the attractor is considered as a node and the links between the nodes are constructed by the distance of the points of the attractor. The generated network inherits the underlying structure and dynamics (deterministic chaos or randomness) of the system. It is suggested [22] that by studying the topology of the network and calculating the degree distribution, we can discriminate chaotic from random time series.

In order to assess the efficiency of visibility and phase space reconstruction methods to discriminate chaos from randomness, we present the methods in Section 2. Then, we review previous relevant work in Section 3. In Section 4, we obtain the degree distribution for each generated network. The meaning of the results is presented in Section 5, and our conclusions in Section 6.

## 2. Networks from Time Series

### 2.1. Visibility Graphs (VG)

Visibility graph (VG) algorithms have gained attention since their introduction by Lacasa et al. in 2008 [17]. Several variations of the original VG algorithm have been proposed utilizing geometric and ordering criteria [14,15,23]. More specifically, connections (edges) are established between the time series values (nodes) using visibility lines [12,24,25]. Visibility graph theory has been used in different areas such as economics [26,27,28], geology [29,30,31], traffic problems [32], tourism [33], the diagnose of Alzheimer’s disease, and biology [34,35,36]. Extensive theory can be found in the literature on Visibility Graphs, containing details of their properties and all the different variations of the method [14,15,23]. In the following, we will briefly present three variants of VGs.

#### 2.1.1. Natural Visibility Graph

The first of the three VG algorithms is the Natural Visibility Graph (NVG), introduced by Lacasa et al. in (2008) [17]. The graph is constructed by connecting points in the time series that are “visible” to each other. A point is considered visible if there are no other points with higher values that block the line of sight between them.

Let ${\left(x_{i}\right)}_{i=1,\ldots ,N}$ with $x_{i}=x\left(t_{i}\right)$ be a time series of N real data. A Natural Visibility Graph is obtained by mapping a time series onto a network according to the following visibility criterion: two arbitrary data $\left(t_{i},x_{i}\right)$ and $\left(t_{j},x_{j}\right)$ in the time series have visibility, if any other data $\left(t_{k},x_{k}\right)$ such that $t_{i}<t_{k}<t_{j}$ fulfills [17]:

(1) $$x_{k}<x_{i}+\left(t_{k}-t_{i}\right)\frac{x_{j}-x_{i}}{t_{j}-t_{i}}$$

which can be written as [14],

(2) $$\frac{x_{i}-x_{k}}{t_{k}-t_{i}}>\frac{x_{i}-x_{j}}{t_{j}-t_{i}}$$

The NVG was claimed to be planar (embeddable in a 2D surface with no overlapping edges) by construction [37,38] without proof. Later, it was stated that this is not true in general [39], again without proof. Below, we give a specific counterexample demonstrating that NVGs are not planar.

**Proposition** **1.**  *There exist non-planar NVGs.*

**Figure 1:**
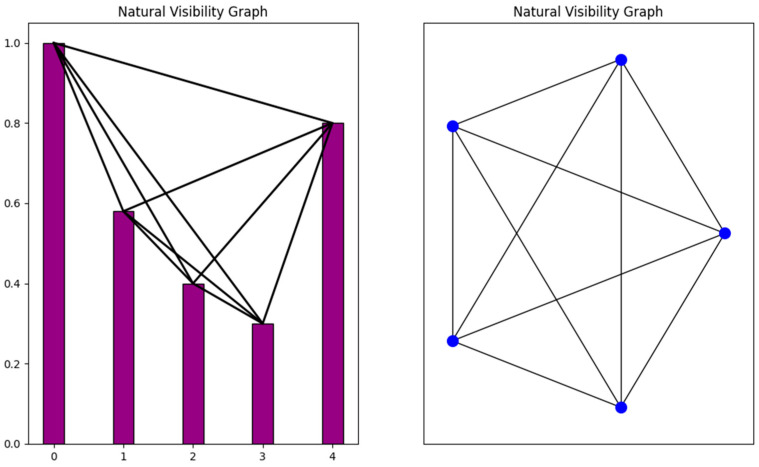
The time series used in the proof of Proposition 1 is presented with the corresponding Natural Visibility Graph. The resulting graph is the graph $K_{5}$ which is non-planar.

**Proof.**  We construct the following non-planar NVG. Consider the time series $\left(1.0,\;0.58,\;0.40,\;0.30,\;0.8\right).$ According to the visibility criterion (Equations (1) and (2)), a Natural Visibility Graph is generated (Figure 1). All planar graphs with N≥3 nodes and E edges satisfy the inequality:

(3) E≤3N−6

Inequality $\left(2\right)$ is a corollary of the Euler’s Formula (1750) [40].

The generated NVG is a complete graph with 5 nodes and 10 edges, as illustrated in Figure 1. It is obvious that the generated NVG does not satisfy inequality (3); therefore, it is not planar. This graph is precisely the graph $K_{5}$. Kuratowski proved in 1930 that a graph is planar if and only if it does not contain a subdivision of the graph $K_{5}$ or a subdivision of the graph $K_{3,3}$ [40,41]. □

#### 2.1.2. Horizontal Visibility Graph

In 2009, Luque et al. suggested a modified version of the NVG algorithm, using a simplified criterion of horizontal visibility to transform time series data into a complex network representation. Two arbitrary data $\left(t_{i},x_{i}\right)$ and $\left(t_{j},x_{j}\right)$ are connected in a horizontal visibility graph, if and only if for all $t_{k}$ with $t_{i}<t_{k}<t_{j}$ [42]:

(4) $$x_{k}<\min\left\{x_{i},x_{j}\right\}$$

**Figure 2:**
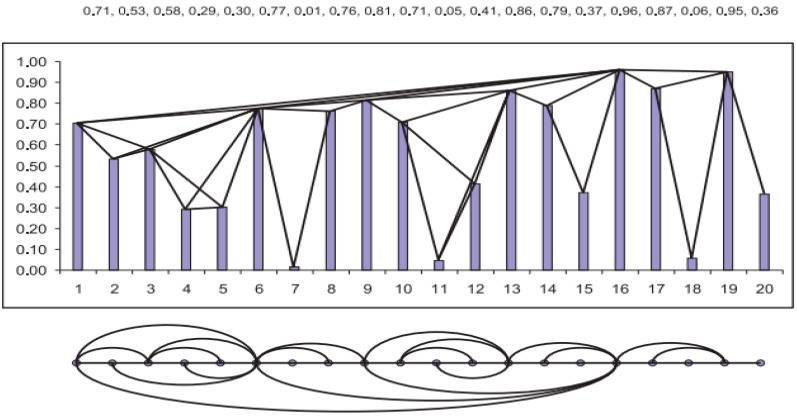

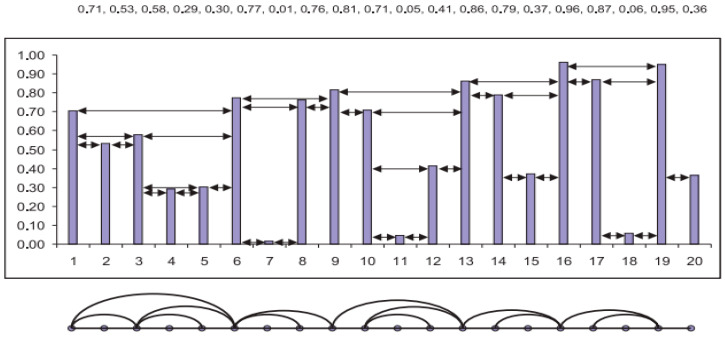
The first row illustrates how a time series is transformed into a network using the natural visibility algorithm, while the second row depicts the same time series transformed using the horizontal visibility algorithm. Notice that the HVG is a subgraph of NVG [23].

If two nodes satisfy Equation (4), then the property of horizontal visibility is fulfilled. Moreover, Equation (3) is also fulfilled; thus, the nodes possess the property of natural visibility as well. Therefore, the degree of the HVG nodes will never be greater than the degree of the corresponding NVG nodes, further implying that the HVG of a given time series is always a subgraph of its NVG [12,14]. The difference between the two methods is illustrated in Figure 2.

#### 2.1.3. Limited Penetrable Horizontal Visibility Graph

**Figure 3:**
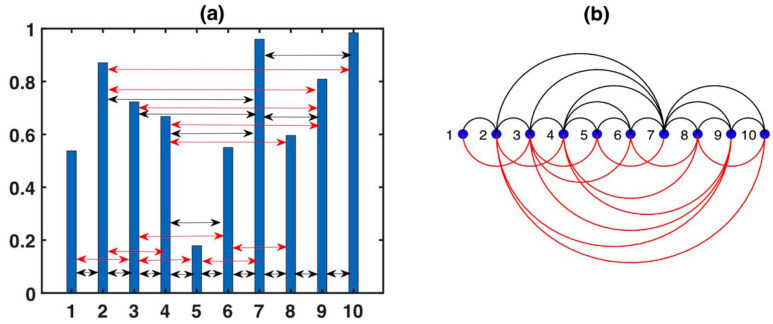
Example of (**a**) a time series with 10 data values and (**b**) its corresponding LPHVG with ρ = 1, where every node corresponds to a time series data. The limited penetrable horizontal visibility lines between data points define the links connecting nodes in the graph. Black lines generate the HVG, and red lines are those added to generate the LPHVG for ρ = 1 [44].

The LPHVG is an enhancement of the HVG. By setting the limited penetrable distance to ρ, a link between two nodes exists if the number of in-between nodes that block the horizontal line is at most ρ [43,44,45,46,47]. If ρ=0, LPHVG degenerates into HVG, but if ρ≠0, there are more connections between any two nodes in LPHVG than in HVG. In Figure 3, we can see the new established connections (red lines) when inferring the LPHVG on the HVG with a limited penetrable distance ρ=1. In general, the LPHVG is denser compared to the corresponding HVG [43]. The limited penetrable horizontal visibility graph inherits many properties of the corresponding horizontal visibility graph, e.g., it is connected and invariant under all affine transformations of the series data [17,42]. However, the HVG planarity property is not inherited to the corresponding LPHVG:

**Proposition** **2.**  *Although all HVG are planar, there exist non-planar LPHVGs.*

**Figure 4:**
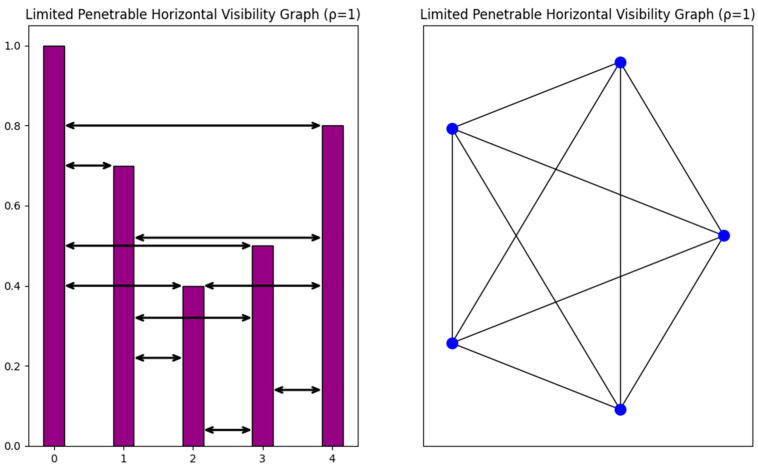
The time series used in the proof of Proposition 2 is presented with the corresponding LPHVG. The resulting graph is the graph $K_{5}$, which is non-planar.

**Proof.**  All HVGs are known to be planar. More specifically, a graph is an HVG if and only if it is outerplanar and has a Hamilton path [48]. We construct the following non-planar LPHVG. Consider the time series $\left(1.0,\;0.7,\;0.40,\;0.50,\;0.8\right).$ For limited penetrable distance ρ=1, the generated graph Figure 4 is not planar as it is a $K_{5}$ graph which is non-planar as discussed in the proof of Proposition 1. Moreover, for the limited penetrable distance ρ≥1, the corresponding LPHVGs are also non-planar since all of them essentially include as a subgraph the LPHVG with ρ=1 which is the non-planar graph $K_{5}$. □

### 2.2. Phase Space Reconstruction Graphs (PSRG)

Reconstructing the phase space of a dynamical system from a time series was introduced by Takens in 1981 [49]. According to this method, for a time series ${\left(x_{i}\right)}_{i=0,1,\ldots ,N}$, where N is the sampling size, we choose a delay time τ. Then, the vector point within the m-dimensional phase space can be represented as follows:

(5) $$X_{k}=\left\{x\left(k\right),x\left(k+\tau \right),x\left(k+2\tau \right),\ldots ,x\left(k+\left(m-1\right)\tau \right)\right\}$$

where k=1,…,M and M=N−(m−1)τ, represent all the vector points of the phase space reconstruction.

In order to construct the complex network, the distances between each pair of vector-points (in the delayed time series) are used as weights between nodes, with the vector-points serving as the nodes themselves. The distance between vector-points in phase space is defined as:

(6) $$d_{ij}=\sum_{n=1}^{m}\left\|X_{i}\left(n\right)-X_{j}\left(n\right)\right\|$$

where $X_{i}(n)=z(i+(n-1)\tau )$ and $X_{j}(n)=z(j+(n-1)\tau )$ is the n^th^ element of $\bar{X_{i}}$ and $\bar{X_{j}}$, m being the embedding dimension, and τ the delay time. In this way, a fully connected weighted network has been constructed. Since this network may contain redundant information, it can be converted into its unweighted counterpart by setting a threshold on the weights. By selecting a threshold $r_{c}$, the distance matrix $D=(d_{ij})$ gives rise to the adjacency matrix $A=\left(a_{ij}\right)$:

(7) $$a_{ij}=\left\{\begin{array}{c} 1,\;d_{ij}\leq r_{c}\\ 0,\;d_{ij}\geq r_{c} \end{array}\right.$$

To determine the optimal threshold, we examine the density of the network. The selected threshold $r_{c}$ corresponds to the maximum value on the plot of the derivative of density versus the threshold [22].

## 3. Signature of Chaos in Networks Associated with Time Series

### 3.1. Signature of Chaos in Visibility Graphs

Lacasa et al. in 2008 demonstrated the applicability of the Natural Visibility Graph (NVG) to a wide range of time series, including chaotic ones [17]. It was also found that the structure of the time series is mapped in the resulting graph topology. Specifically, periodic time series convert into regular graphs, random series into random graphs, and fractal series convert into scale-free graphs, indicating that power law degree distributions are related to fractality.

Luque et al. in 2009 proved that any uncorrelated random series maps to a graph with an exponential degree distribution of the shape [42]:

(8) $$P\left(k\right)=\frac{1}{3}{\left(\frac{2}{3}\right)}^{k-2},k=2,3,4,\ldots$$

Lacasa and Toral in 2010 tried to use the horizontal visibility graph to characterize and distinguish between stochastic and chaotic processes [16]. They suggested that in every case we get a network with exponential degree distribution $P(k)\sim e^{-\lambda k}$, where the value of λ indicates the type of process generating the time series. In specific, they claim that for $\lambda <\ln\left(\frac{3}{2}\right)$ we have a chaotic process, whereas $\lambda >\ln\left(\frac{3}{2}\right)$ corresponds to a correlated stochastic process. The boundary value $\lambda_{c}=\ln\left(\frac{3}{2}\right)$ corresponds to the uncorrelated case.

However, Ravetti et al. in 2014 found some examples of chaotic time series where $\lambda >\lambda_{c}$ and stochastic time series where $\lambda <\lambda_{c}$, indicating that the above rule does not hold in a strict way [50]. Also, Zhang et al. in 2017 considered time series generated by auto-regressive (AR) processes and provided some examples supporting that correlated stochastic time series are characterized by $\lambda >\lambda_{c}$, slowly tending to an asymptotic value of $\ln\left(\frac{3}{2}\right)$ for weak correlations. Moreover, they also found some peculiar results indicating that $\lambda_{c}$ should not be interpreted as a general critical value separating chaos from noise [51].

Although some time series were found for which the proposed λ—criterion fails to successfully characterize them as chaotic or stochastic, several recent (post–2020) papers use it to discriminate chaotic from stochastic time series. Specifically, in [52], the λ—exponent criterion is applied to the HVG [16] to characterize magnetic fluctuation time series obtained from PIC simulations. Also, the time series of streamflow [53], air traffic flow [54], cryptocurrencies price [55], and the air mean temperature [56] are characterized as chaotic or stochastic depending on the exponent λ.

Similar results have been obtained for LPHVG (Section 2.1.3). Wang et al. in 2018 found that this graph can discriminate chaos from uncorrelated randomness [44]. In specific, they showed that random time series map on an LPHVG with an exponential degree distribution $P\left(k\right)\sim \exp\left[-\lambda \left(k-2\rho -2\right)\right]$ with $\lambda =\ln\left[\frac{2\rho +3}{2\rho +2}\right]$, ρ=0, 1, 2,… and k=2ρ+2,2ρ+3,…. The degree distribution was found to be independent of the probability distribution from which the series was generated. It was also found that degree distribution corresponding to chaotic systems can be approximated by the exponential function $P(k)\sim \exp\left(-\hat{\lambda }k\right)$, with $\hat{\lambda }\neq \lambda =\ln\left[\frac{2\rho +3}{2\rho +2}\right]$. Parameter $\hat{\lambda }$ also indicates the boundary between random and chaotic series; thus, it can be used to distinguish randomness from chaos.

### 3.2. Signature of Chaos in Phase Space Reconstruction Graphs

Zhongke Gao and Ningde Jin in 2009 [22] proposed the phase space graph method and found that the constructed network inherits the main properties of the time series in its structure. Specifically, periodic series are mapped into regular networks, noisy series into random networks, and chaotic series (typically) into small world and scale-free networks [22].

Similarly, the phase space coarse graining algorithm [57] converts the time series into a directed and weighted complex network. It was also claimed that periodic series convert into regular networks, while random series convert into random networks and chaotic series into scale-free networks [57].

Therefore, both PSRG and coarse grain PSRG constructions are expected to allow the distinction between chaos and randomness [22,57].

## 4. Results

As mentioned in Section 1, chaos is expected to be statistically indistinguishable from randomness. In order to discriminate chaos from randomness, we decided to map the time series into complex networks and investigate whether the discrimination is possible by comparing the associated networks.

We selected three different time series of 1000 values each, coming from three different systems, namely, the Torus Automorphisms, the Lorenz System, and a random sequence. The chaotic Torus Automorphisms are obtained using Equations (9)–(12), with parameters h=1, a=2, b=1, and h=10, a=1, b=1000, the Lorenz System is obtained through the dependence of variable x through the three Lorenz equations, with parameters σ=10, ρ=28, $\beta =\frac{8}{3}\;$, and the Random Sequence is obtained from the Standard Gaussian Distribution (mean 0 and standard deviation 1). For each time series, we constructed four associated graphs, namely NVG, HVG, LPHVG, and PSRG, and computed their degree distributions which are presented in Section 4.1, Section 4.2 and Section 4.3. Most studies claim that the discrimination of chaos from randomness follows by observing the degree distributions [16,17,22,42,44,57]. We shall examine whether the degree distributions obey a power law distribution $P(k)\sim k^{-\gamma }$, or an exponential distribution $P(k)\sim e^{-\lambda k}$. These distributions are claimed to correspond to chaos and randomness (Section 3.1 and Section 3.2).

We present the degree distributions in a lin–lin plot, and in a log–log plot in order to identify power law and in a lin–log plot in order to identify exponential law. The slope (exponent of the distribution) is computed with the least square method. In the case of the LPHVG, we select two values of limited penetrable distance, namely ρ=1 and ρ=2. In the case of PSRG, we use the technique proposed by Zhongke Gao and Ningde Jin to find the optimal delay time, τ [22], and the technique proposed by Matthew B. Kennel and Reggie Brown to determine the optimal embedding dimension, m (False Nearest Neighbor algorithm, FNN) [58]. The FNN method requires the selection of certain parameters in order to find the optimal value of m, so we decided to use Cao’s method as well [59].

### 4.1. Results for Torus Automorphisms

The Torus Automorphisms are transformations of the 2—Torus $Y=\left[0,1\right)\times \left[0,1\right)$ given by the formula:

(9) $$S:Y\to Y:\;\left(\begin{array}{c} x_{n+1}\\ y_{n+1} \end{array}\right)=A\left(\begin{array}{c} x_{n}\\ y_{n} \end{array}\right)\left(mod1\right),n\in Z$$

where $A=\left(\begin{array}{cc} a & b\\ c & d \end{array}\right)$ a 2×2 matrix with a,b,c,d∈Z and $\det\left(A\right)=1$.

The matrix element c of A can be expressed in terms of the entropy production rate h as follows [60]:

(10) $$A=\left(\begin{array}{cc} a & b\\ \frac{ad-1}{b} & d \end{array}\right),b\neq 0,d>2-a$$

The entropy production h can be expressed in terms of the positively Lyapunov exponent as:

(11) $$\;h=\log_{2}\lambda_{+}=\log_{2}\frac{\left(a+d\right)+\sqrt{{\left(a+d\right)}^{2}-4}}{2}=\log_{2}\frac{tr\left(A\right)+\sqrt{{\left(tr\left(A\right)\right)}^{2}-4}}{2}$$

Therefore:

(12) $$A=\left(\begin{array}{cc} a & b\\ \frac{a\left(2^{h}+2^{-h}-a\right)-1}{b} & 2^{h}+2^{-h}-a \end{array}\right),h>0$$

Entropy production is defined by Kolmogorov [6]. Using the above formulas, we generated 6 chaotic time series (from the 2—Torus automorphisms) of 1000 values each and with entropy production for each of them: h=1 and h=10. We obtained 12 chaotic time series, with the least chaotic ones having an entropy production of 1, which goes up to 10. In the paper, we present two chaotic time series of the variable y, with entropy production h=1 and h=10.

#### 4.1.1. Natural Visibility Graph of Torus Automorphisms

**Figure 5:**
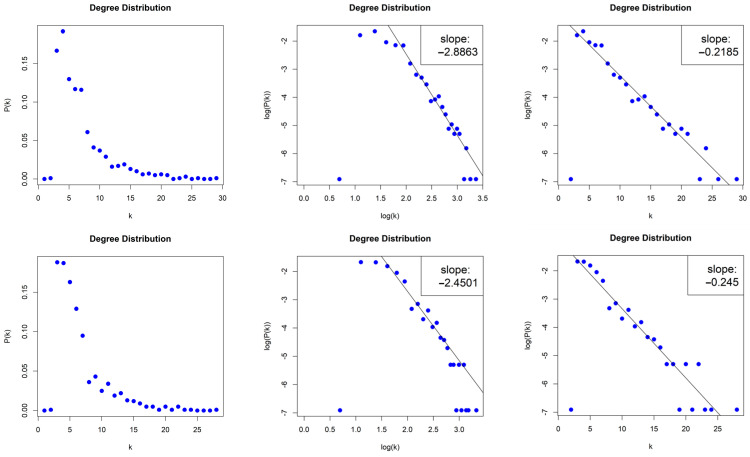
Degree distribution of the Natural Visibility Graphs of two Torus Automorphisms corresponding to the chaotic time series with h=1 (first row) and h=10 (second row), in lin–lin scale (left column), in log–log scale (middle column), and in lin–log scale (right column).

The NVGs generated by the 2D Torus Automorphisms are sparse with density d≈0.0057. For both values of entropy production, we obtained scale-free networks (middle column, Figure 5) with degree distributions that asymptotically follow a power law $P(k)\sim k^{-\gamma }$ and with exponents (slopes) $\gamma_{h=1}=-2.886$ and $\gamma_{h=10}=-2.45$. The error of the least square fit is $R_{h=1}^{2}=0.96$ and $R_{h=10}^{2}=0.95$. The contribution of fat tails is neglected. One may also consider the degree distributions as exponential distribution $P(k)\sim e^{-\lambda k}$ (right column, Figure 5) with exponents (slopes) $\lambda_{h=1}=-0.218$ and $\lambda_{h=10}=-0.245$ with errors $R_{h=1}^{2}=0.96$ and $R_{h=10}^{2}=0.92$.

#### 4.1.2. Horizontal Visibility Graph of Torus Automorphisms

**Figure 6:**
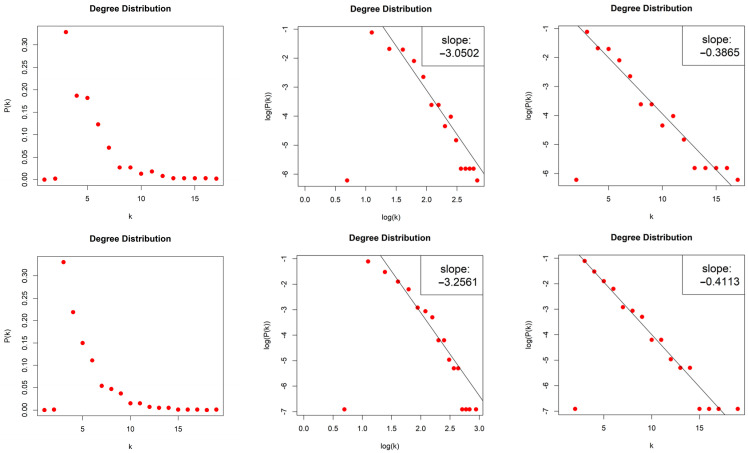
Degree distribution of the Horizontal Visibility Graphs of two Torus Automorphisms corresponding to the chaotic time series with h=1 (first row) and h=10 (second row), in lin–lin scale (left column), in log–log scale (middle column), and in lin–log scale (right column).

The HVGs generated by the 2D Torus Automorphisms are sparse with density d≈0.004. For both values of entropy production, we obtained scale-free networks (middle column, Figure 6) with degree distributions that asymptotically follow a power law $P(k)\sim k^{-\gamma }$ and with exponents (slopes) $\gamma_{h=1}=-3.05$ and $\gamma_{h=10}=-3.256$. The error of the least square fit is $R_{h=1}^{2}=0.93$ and $R_{h=10}^{2}=0.96$. The contribution of fat tails is neglected. One may also consider the degree distributions as exponential distribution $P(k)\sim e^{-\lambda k}$ (right column, Figure 6) with exponents (slopes) $\lambda_{h=1}=-0.386$ and $\lambda_{h=10}=-0.411$ with errors $R_{h=1}^{2}=0.96$ and $R_{h=10}^{2}=0.97$.

#### 4.1.3. Limited Penetrable Horizontal Visibility Graph of Torus Automorphisms

**Figure 7:**
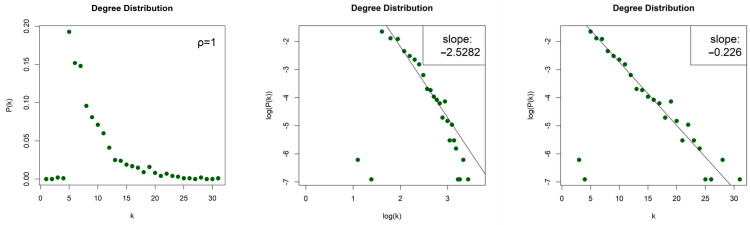

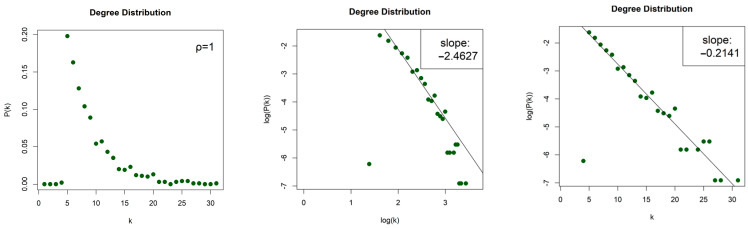
Degree distribution of the Limited Penetrable Horizontal Visibility Graphs with limited penetrable distance ρ = 1 of two Torus Automorphisms corresponding to the chaotic time series with h=1 (first row) and h=10 (second row), in lin–lin scale (left column), in log–log scale (middle column), and in lin–log scale (right column).

**Figure 8:**
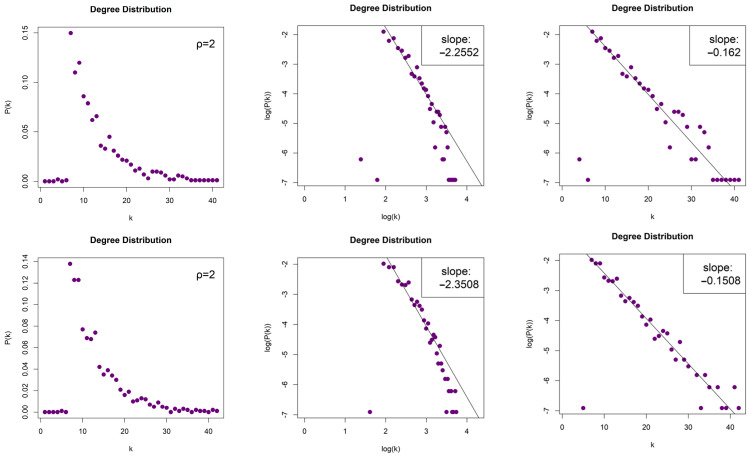
Degree distribution of the Limited Penetrable Horizontal Visibility Graphs with limited penetrable distance ρ = 2 of two Torus Automorphisms corresponding to the chaotic time series with h=1 (first row) and h=10 (second row), in lin–lin scale (left column), in log–log scale (middle column), and in lin–log scale (right column).

The LPHVGs generated by the 2D Torus Automorphisms are sparse for both values of ρ, with density $d_{\rho =1}\approx 0.008$ and $d_{\rho =2}\approx 0.012$. For both values of entropy production, we obtained scale-free networks (middle column, Figure 7 for ρ = 1 and Figure 8 for ρ = 2) with degree distributions that asymptotically follow a power law $P(k)\sim k^{-\gamma }$ and with exponents (slopes) $\gamma_{\rho =1}=-2.528$ and $\gamma_{\rho =2}=-2.255$ for h=1, as well as $\gamma_{\rho =1}=-2.462$ and $\gamma_{\rho =2}=-2.35$ for h=10. The error of the least square fit is $R_{\rho =1}^{2}=0.94$ and $R_{\rho =2}^{2}=0.93$ for h=1, and $R_{\rho =1}^{2}=0.96$ and $R_{\rho =2}^{2}=0.93$ for h=10. The contribution of fat tails is neglected. One may also consider the degree distributions as exponential distribution $P(k)\sim e^{-\lambda k}$ (right column, Figure 7 for ρ = 1 and Figure 8 for ρ = 2) with exponents (slopes) exponent $\lambda_{\rho =1}=-0.226$ and $\lambda_{\rho =2}=-0.162$, with errors $R_{\rho =1}^{2}=0.96$ and $R_{\rho =2}^{2}=0.97$ for h=1, as well as $\lambda_{\rho =1}=-0.214$ and $\lambda_{\rho =2}=-0.15$, with $R_{\rho =1}^{2}=0.96$ and $R_{\rho =2}^{2}=0.95$ for h=10.

#### 4.1.4. Phase Space Reconstruction Graph of Torus Automorphisms

**Figure 9:**
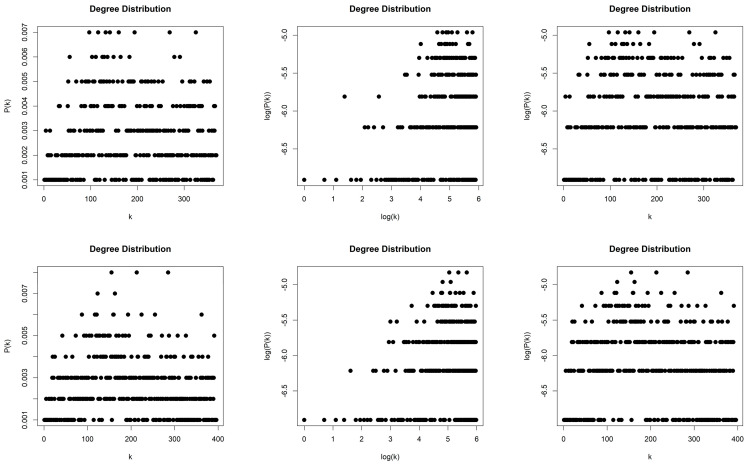
Degree distribution of the Phase Space Reconstruction Graphs of two Torus Automorphisms corresponding to the chaotic time series with h=1 (first row) and h=10 (second row), in lin–lin scale (left column), in log–log scale (middle column), and in lin–log scale (right column).

The PSRGs generated by the 2D Torus Automorphisms are dense with density $d_{h=1}\approx 0.4$ and $d_{h=10}\approx 0.46$. The parameters chosen to generate the network are τ=1, m=2, and $r_{c}=0.62$ for h=1 and τ=1, m=2, $r_{c}=0.56$ for h=10. As illustrated from the degree distribution in Figure 9, the distribution follows neither a power law nor an exponential law.

### 4.2. Results for the Lorenz System

#### 4.2.1. Natural Visibility Graph of the Lorenz System

**Figure 10:**
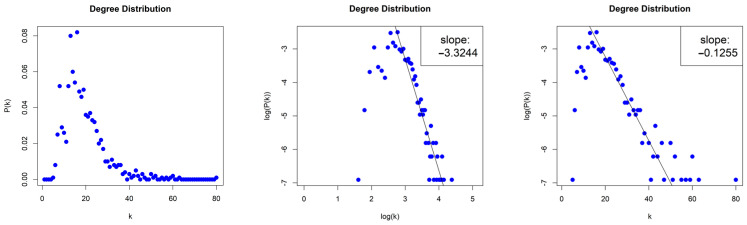
Degree distribution of the Natural Visibility Graph of the Lorenz System in lin–lin scale (left column), in log–log scale (middle column), and in lin–log scale (right column).

The NVG generated by the Lorenz System is sparse with density d≈0.018, and scale-free (middle column, Figure 10) with degree distribution that follows an asymptotic power law $P(k)\sim k^{-\gamma }$ for $\log\left(k\right)\geq \log\left(12\right)$, and with exponent (slope) γ=−3.324. The error of the least square fit is $R^{2}=0.9$. The contribution of fat tails is neglected. One may also consider the degree distribution as exponential distribution $P(k)\sim e^{-\lambda k}$ for k≥12, (right column, Figure 10) with exponent (slope) λ=−0.126 and error $R^{2}=0.94$.

#### 4.2.2. Horizontal Visibility Graph of the Lorenz System

**Figure 11:**
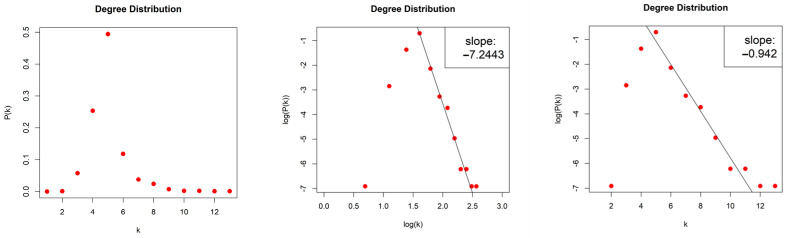
Degree distribution of the Horizontal Visibility Graph of the Lorenz System in lin–lin scale (left column), in log–log scale (middle column), and in lin–log scale (right column).

The HVG generated by the Lorenz System is sparse with density d≈0.004, and scale-free (middle column, Figure 11) with degree distribution that asymptotically follows a power law $P(k)\sim k^{-\gamma }$ and with exponent (slope) γ=−7.244. The error of the least square fit is $R^{2}=0.98$. One may also consider the degree distribution as exponential distribution $P(k)\sim e^{-\lambda k}$ (right column, Figure 11) with exponent (slope) λ=−0.942 and error $R^{2}=0.97$.

#### 4.2.3. Limited Penetrable Horizontal Visibility Graph of the Lorenz System

**Figure 12:**
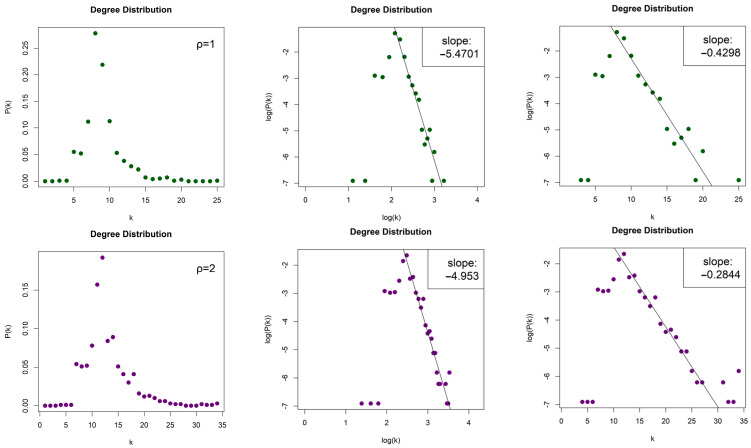
Degree distribution of the Limited Penetrable Horizontal Visibility Graphs with limited penetrable distance ρ = 1 (first raw) and ρ = 2 (second raw) of the Lorenz System in lin–lin scale (left column), in log–log scale (middle column), and in lin–log scale (right column).

The LPHVGs generated by the Lorenz System are sparse for both values of ρ, with density $d_{\rho =1}\approx 0.008$ and $d_{\rho =2}\approx 0.012$. For both values of ρ, we obtained scale-free networks (middle column, Figure 12) with degree distributions that follow an asymptotic power law $P\left(k\right)\sim k^{-\gamma }$ for $\log\left(k\right)\geq \log\left(6\right)$ and $\log\left(k\right)\geq \log\left(9\right)$, for ρ=1 and ρ=2, respectively, and with exponents (slopes) $\gamma_{\rho =1}=-5.47$ and $\gamma_{\rho =2}=-4.953$. The error of the least square fit is $R_{\rho =1}^{2}=0.94$ and $R_{\rho =2}^{2}=0.96$. One may also consider the degree distributions as exponential distribution $P(k)\sim e^{-\lambda k}$ for k≥6 and k≥9, (right column, Figure 12) with exponents (slopes) $\lambda_{\rho =1}=-0.43$ and $\lambda_{\rho =2}=-0.284$ and errors $R_{\rho =1}^{2}=0.93$ and $R_{\rho =2}^{2}=0.96$.

#### 4.2.4. Phase Space Reconstruction Graph of the Lorenz System

**Figure 13:**
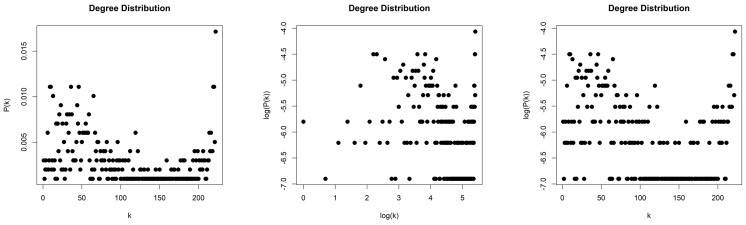
Degree distribution of the Phase Space Reconstruction Graph of the Lorenz System in lin–lin scale (left column), in log–log scale (middle column), and in lin–log scale (right column).

The PSRG generated by the Lorenz System is dense with density d≈0.88. The parameters chosen to generate the network are τ=2, m=3, $r_{c}=44$. As illustrated from the degree distribution in Figure 13, the distribution follows neither a power law nor an exponential law.

### 4.3. Results for the Random Sequence with Gaussian Distribution

#### 4.3.1. Natural Visibility Graph of the Random Sequence

**Figure 14:**
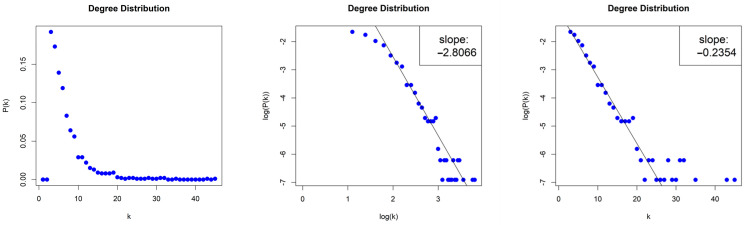
Degree distribution of the Natural Visibility Graph of the Random Sequence in lin–lin scale (left column), in log–log scale (middle column), and in lin–log scale (right column).

The NVG generated by the Random Sequence is sparse with density d≈0.0059. We obtained a scale-free network (middle column, Figure 14) with degree distribution that asymptotically follows a power law $P(k)\sim k^{-\gamma }$ and with exponent (slope) γ=−2.807. The error of the least square fit is $R^{2}=0.95$. The contribution of fat tails is neglected. One may also consider the degree distribution as exponential distribution $P(k)\sim e^{-\lambda k}$ (right column, Figure 14) with exponent (slope) λ=−0.235 and with error $R^{2}=0.97$.

#### 4.3.2. Horizontal Visibility Graph of the Random Sequence

**Figure 15:**
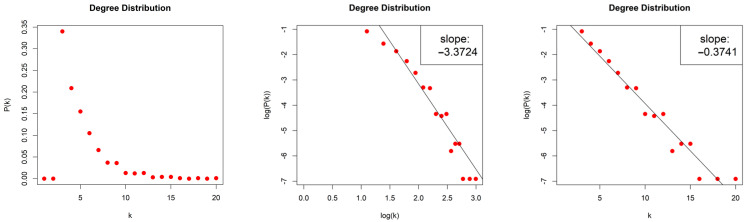
Degree distribution of the Horizontal Visibility Graph of the Random Sequence in lin–lin scale (left column), in log–log scale (middle column), and in lin–log scale (right column).

The HVG generated by the Random Sequence is sparse with density d≈0.004. We obtained a scale-free network (middle column, Figure 15) with degree distribution that asymptotically follows a power law $P(k)\sim k^{-\gamma }$ and with exponent (slope) γ=−3.372. The error of the least square fit is $R^{2}=0.93$. One may also consider the degree distribution as exponential distribution $P(k)\sim e^{-\lambda k}$, (right column, Figure 15) with exponent (slope) λ=−0.374 and error $R^{2}=0.95$.

#### 4.3.3. Limited Penetrable Horizontal Visibility Graph of the Random Sequence

**Figure 16:**
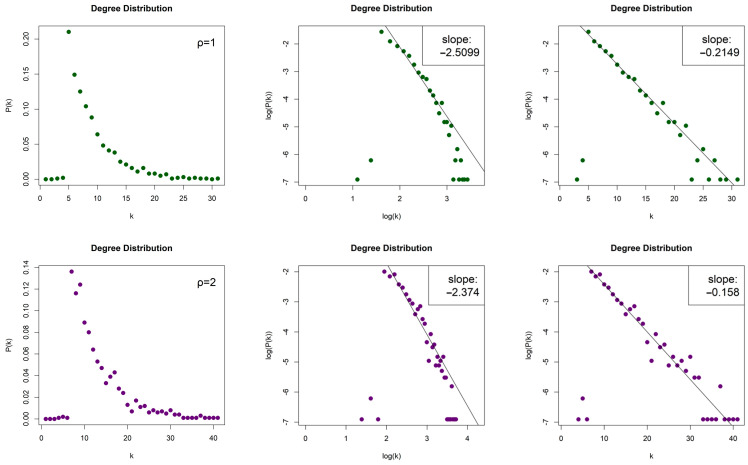
Degree distribution of the Limited Penetrable Horizontal Visibility Graphs with limited penetrable distance ρ = 1 (first raw) and ρ = 2 (second raw) of the Random Sequence, in lin–lin scale (left column), in log–log scale (middle column), and in lin–log scale (right column).

The LPHVGs generated by the Random Sequence are sparse for both values of ρ, with density $d_{\rho =1}\approx 0.008$ and $d_{\rho =2}\approx 0.012$. For both values of ρ, we obtained scale-free networks (middle column, Figure 16) with degree distributions that asymptotically follow a power law $P\left(k\right)\sim k^{-\gamma }$, with exponents (slopes) $\gamma_{\rho =1}=-2.51$ and $\gamma_{\rho =2}=-2.374$. The error of the least square fit is $R_{\rho =1}^{2}=0.95$ and $R_{\rho =2}^{2}=0.89$. The contribution of fat tails is neglected. One may also consider the degree distributions as exponential distribution $P(k)\sim e^{-\lambda k}$, (right column, Figure 16) with exponents (slopes) $\lambda_{\rho =1}=-0.215$ and $\lambda_{\rho =2}=-0.158$ and with errors $R_{\rho =1}^{2}=0.98$ and $R_{\rho =2}^{2}=0.94$.

#### 4.3.4. Phase Space Reconstruction of the Random Sequence

**Figure 17:**
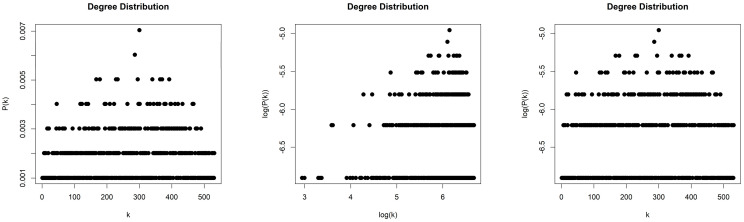
Degree distribution of the Phase Space Reconstruction Graph of the Random Sequence in lin–lin scale (left column), in log–log scale (middle column), and in lin–log scale (right column).

The PSRG generated by the Random Sequence is dense with density d≈0.43. The parameters chosen to generate the network are τ=1, m=7, and $r_{c}=7.6$. As illustrated from the degree distribution in Figure 17, the distribution follows neither a power law nor an exponential law.

## 5. Meaning of the Results

### 5.1. Visibility Graphs

The visibility methods [17,42,44] concluded with some robust results, generating sparse, topologically similar networks, with the degree distribution obeying most of the cases in terms of both power and exponential law. The chaotic 2D Torus Automorphisms and the Random Sequence generated pure scale-free networks with similar, indistinguishable slopes (exponents of the power/exponential law) for both log–log and lin–log plots. Similar but not identical results were found for the network constructed by the Lorenz System, for which degree distribution follows both an asymptotic power law and an exponential law, for k≥12. The slopes calculated were not identical with the other two systems studied. In summary, no distinguishment is found among the values of the exponents of the power or exponential law for the chaotic systems and the Random Sequence.

### 5.2. Phase Space Reconstruction Graphs

The PSRGs were a lot denser than the VGs. The proposed methodology for the Phase Space Reconstruction method [22] did not generate scale-free networks as expected for either of the systems studied. In fact, the degree distribution of both the chaotic time series and the Random Sequence is similar, but it cannot be fitted in power or exponential law.

## 6. Concluding Remarks

The purpose of this study is to investigate the extent to which networks generated from time series can distinguish the presence of chaos or randomness in the time series. We examined three representative visibility methods for mapping time series to networks, namely NVG [17], HVG [42], LPHVG [44], and the phase space reconstruction method [22].

It has been suggested that chaotic time series generate networks with power law degree distributions. This has been confirmed in several cases [61]. However, we found that such a distinction is not possible in general. In the case of Phase Space Reconstruction, we found chaotic time series generating non scale-free networks, while for the visibility methods, we found that both chaotic time series and random sequence generate indistinguishable scale-free networks (Section 4 and Section 5).

In the case of the networks constructed using the visibility methods, the degree distribution may also be interpreted as following an exponential law. We demonstrated (Section 5.1) that the λ—exponent criterion applied to HVG (Section 3.1) [16,50,51,52,53,54,55,56] cannot reliably distinguish chaos from randomness since practically indistinguishable exponents are found for both chaotic and random processes. Therefore, the distinction cannot rely only on the λ—exponent criterion. The confirmation of the distinction requires further investigation. Similar conclusions are obtained for the λ—exponent criterion applied to LPHVG (Section 3.1) [44], where we also found that both chaotic and random processes give rise to the same exponents (Section 5.1).

Also, two proofs regarding the VGs are provided. The first one is that there exist non-planar NVGs (Proposition 1) and the second one is that although all HVGs are planar, there exist non-planar LPHVGs (Proposition 2). Both propositions are proved by constructing counter-examples. Although the discussion of planarity is not connected directly to the results of this research, it is a property of the NVG and LPHVG.

We observe a significant difference between VGs and PSRGs. Visibility methods generate scale-free networks for every case studied, with robust and stable results, while the phase space reconstruction method does not give rise to scale-free networks (Section 4 and Section 5). However, although VGs have power law degree distributions, the exponents are indistinguishable (Table 1, Section 5).

Our conclusion is not quite unexpected, as remarked by Ravetti et al. [50] and Zhang et al. [51]. However, the arguments presented in [50,51] referred only to the case of HVG and, specifically, the λ-criterion proposed by Lacasa and Toral [16]. The idea that chaos can be discriminated from randomness via complex networks was discussed without reservation [52,53,54,55,56]. We explored all other cases which claim the possibility of the distinction of chaos from randomness using the degree distribution of the associated VGs and PSRGs.

Concluding, neither method was able to efficiently distinguish between chaos and randomness. Power law degree distributions cannot be considered as a generic feature of chaos. Although the methods we studied are topological, apparently the diagnosis is made with statistical tools and, consequently, no satisfactory distinction can be achieved without additional information.

## Figures and Tables

**Table 1:** The slopes of the log–log and lin–log plots for the VGs and PSRGs indicating power law and exponential representation of the underlying degree distribution.

Time Series Source	NVG Power|Exp log–log|lin–log	HVG Power|Exp log–log|lin–log	LPHVG (ρ = 1) Power|Exp log–log|lin–log	LPHVG (ρ = 2) Power|Exp log–log|lin–log
Torus, h=1	−2.8863|−0.2185	−3.0502|−0.3865	−2.5282|−0.2260	−2.2552|−0.1620
Torus, h=10	−2.4501|−0.2450	−3.2561|−0.4113	−2.4627|−0.2141	−2.3508|−0.1508
Lorenz System	−3.3244|−0.1255	−7.2443|−0.9420	−5.4701|−0.4298	−4.9530|−0.2844
Random Sequence	−2.8066|−0.2354	−3.3724|−0.3741	−2.5099|−0.2149	−2.3740|−0.1580

## Data Availability

The data presented in this study are available upon request from the corresponding author.
